# The Genetic Basis of Hypertriglyceridemia

**DOI:** 10.1007/s11883-021-00939-y

**Published:** 2021-06-19

**Authors:** Germán D. Carrasquilla, Malene Revsbech Christiansen, Tuomas O. Kilpeläinen

**Affiliations:** Novo Nordisk Foundation Center for Basic Metabolic Research, Faculty of Health and Medical Sciences, University of Copenhagen, Mærsk Building, Blegdamsvej 3B, 2200 Copenhagen, Denmark

**Keywords:** Hypertriglyceridemia, Triglycerides, Dyslipidemia, Human genetics, Genetic variant, Monogenic, Polygenic

## Abstract

**Purpose of Review:**

Hypertriglyceridemia is a common dyslipidemia associated with an increased risk of cardiovascular disease and pancreatitis. Severe hypertriglyceridemia may sometimes be a monogenic condition. However, in the vast majority of patients, hypertriglyceridemia is due to the cumulative effect of multiple genetic risk variants along with lifestyle factors, medications, and disease conditions that elevate triglyceride levels. In this review, we will summarize recent progress in the understanding of the genetic basis of hypertriglyceridemia.

**Recent Findings:**

More than 300 genetic loci have been identified for association with triglyceride levels in large genome-wide association studies. Studies combining the loci into polygenic scores have demonstrated that some hypertriglyceridemia phenotypes previously attributed to monogenic inheritance have a polygenic basis. The new genetic discoveries have opened avenues for the development of more effective triglyceride-lowering treatments and raised interest towards genetic screening and tailored treatments against hypertriglyceridemia.

**Summary:**

The discovery of multiple genetic loci associated with elevated triglyceride levels has led to improved understanding of the genetic basis of hypertriglyceridemia and opened new translational opportunities.

## Introduction

Hypertriglyceridemia, defined as plasma triglyceride levels > 1.7 mmol/L (150 mg/dL), affects about 10% of the adult population and it is thus the most common form of dyslipidemia worldwide [1, 2]. Patients with hypertriglyceridemia are at an increased risk of stroke and coronary heart disease [3–6] and, in severe cases, may develop acute pancreatitis [7–9]. It is still debated whether triglycerides directly promote cardiovascular disease or represent a biomarker for the increased levels of atherogenic, triglyceride-rich lipoproteins [10, 11]. Hypertriglyceridemia is often concomitant with other conditions, such as obesity, type 2 diabetes, and the metabolic syndrome, which contribute to the associated morbidity, mortality, and health care costs [9].

The clinical diagnosis of hypertriglyceridemia is based on fasting triglyceride levels, but non-fasting plasma triglyceride levels can also be applied [8, 11–13]. There is no global consensus on the threshold values for classification of hypertriglyceridemia. For the purposes of the present review, we will apply the guidelines of the European Atherosclerosis Society and the European Society of Cardiology that categorize hypertriglyceridemia into mild-to-moderate (1.7–10 mmol/L) and severe (> 10 mmol/L) types [11]. Mild-to-moderate hypertriglyceridemia indicates a triglyceride level at which cardiovascular disease risk is increased, and severe hypertriglyceridemia indicates an increased risk of acute pancreatitis [11]. Severe hypertriglyceridemia is uncommon, observed in ~0.01% of the general population [14] and ~1–2% of all hypertriglyceridemic adults [1, 2].

**Fig. 1:**
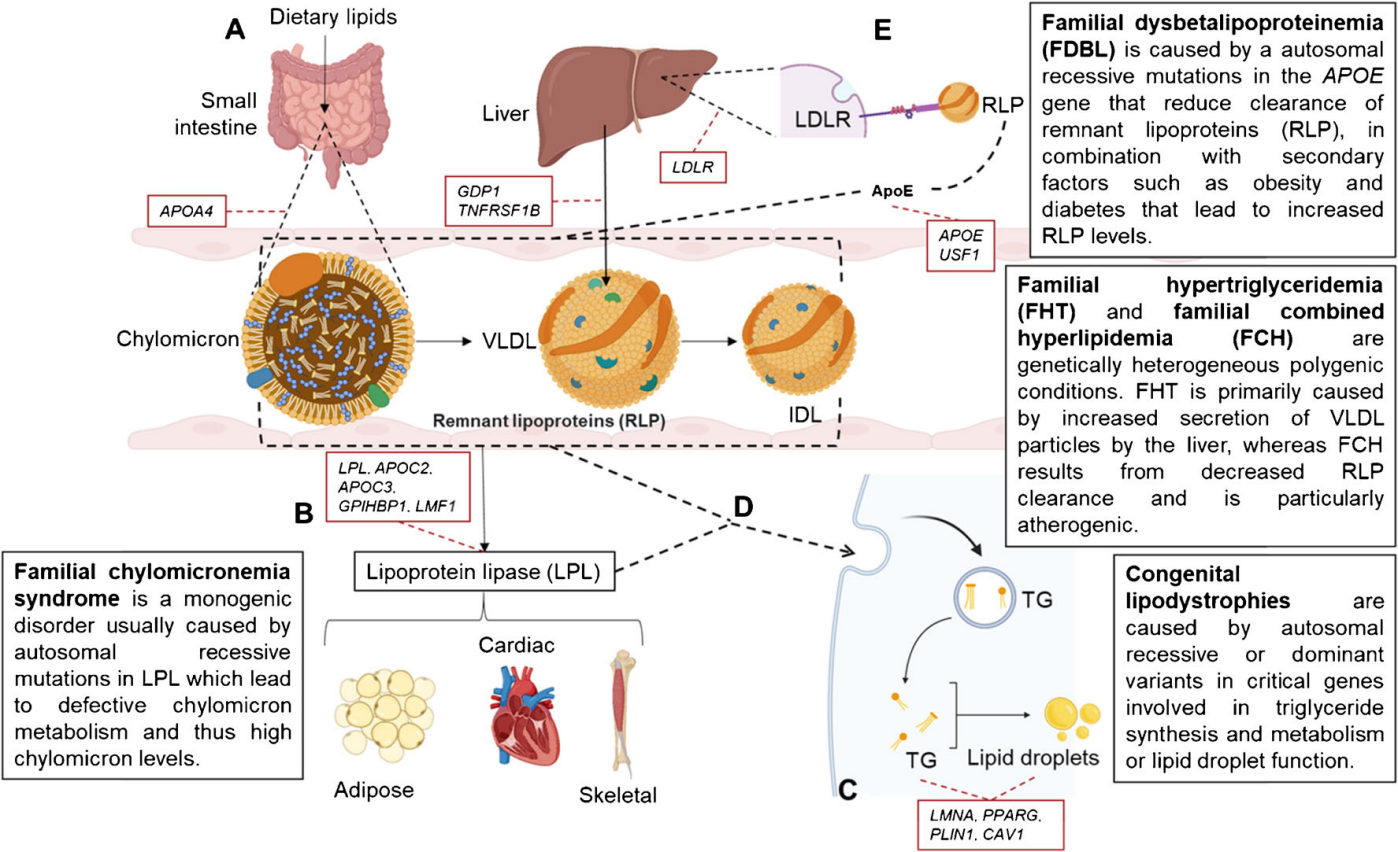
Genetic mechanisms implicated in hypertriglyceridemia. **A** Dietary triglycerides are absorbed from the small intestine and enter the circulation packed in chylomicrons. The liver synthesizes triglycerides de novo from free fatty acids and packs them into very low-density lipoproteins (VLDLs). **B** The chylomicrons and VLDL deliver the triglycerides to adipose tissue, skeletal muscle, and cardiac muscle where lipoprotein lipase (LPL) cleaves triglycerides. **C** In the adipose tissue, triglycerides are stored within adipocyte lipid droplets. **D** The breakdown of triglycerides by LPL turns chylomicrons and VLDL gradually into remnant lipoproteins (RPLs). **E** Remnant lipoproteins are cleared by the liver via ApoE that facilitates binding of RLP to the LDL receptor (LDLR) on the liver. When RPL levels are high, RLP clearance by the liver reaches saturation and RLP molecules accumulate in plasma. Figure icons were created with BioRender.com

**Fig. 2:**
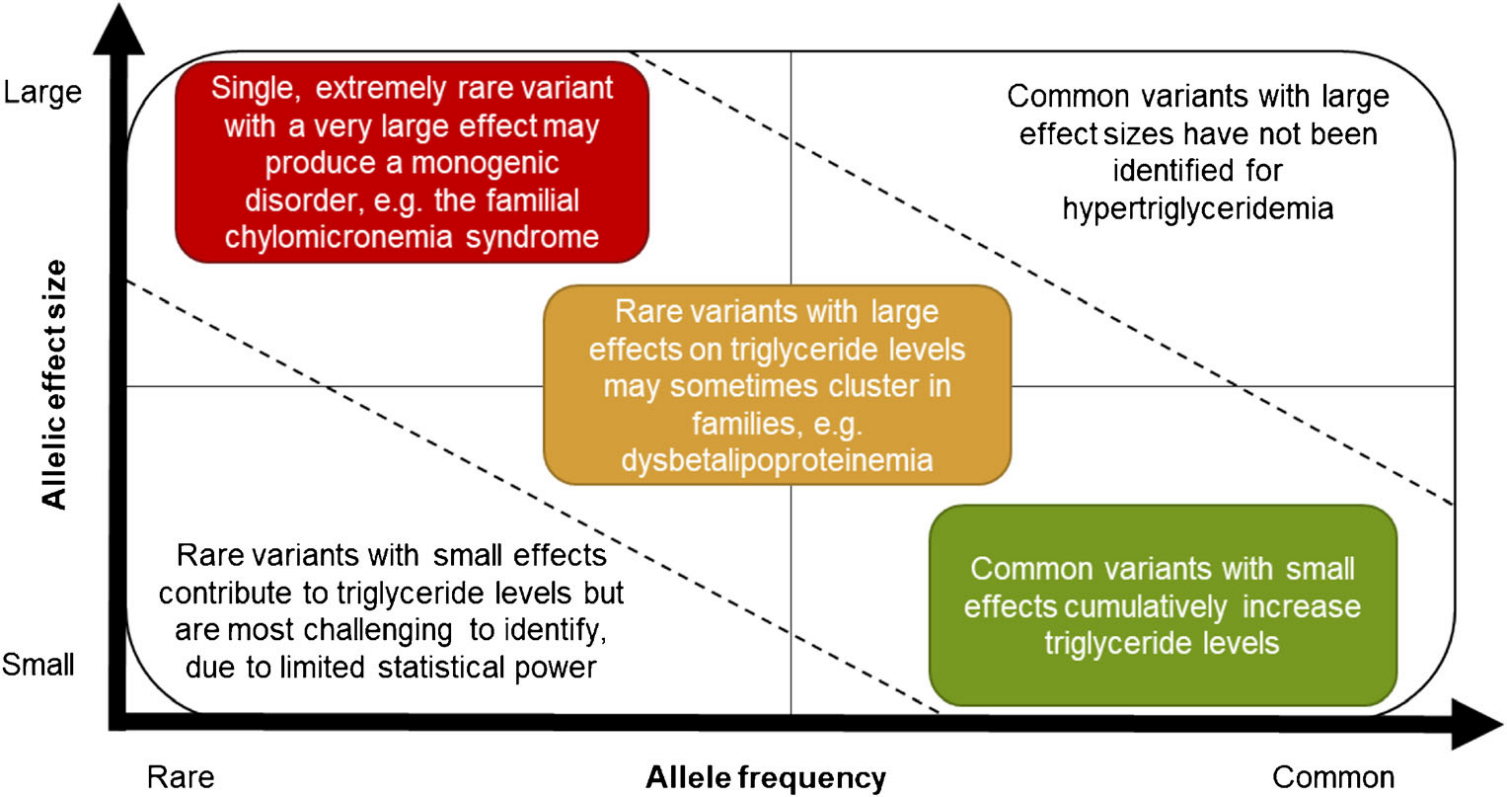
The genetic architecture of hypertriglyceridemia. In very rare, monogenic disorders, a single recessive variant may cause a severe hypertriglyceridemia (*red box*). Rare heterozygous variants with large effects may contribute to the clustering of certain hypertriglyceridemia phenotypes in families (*yellow box*). In most cases of hypertriglyceridemia, the genetic basis is highly polygenic, driven by the cumulative effect of multiple common and rare variants with modest effect sizes (*green box*)

Genetic mechanisms that predispose to hypertriglyceridemia affect the synthesis, metabolism, or clearance of triglycerides, leading to increased levels of triglycerides in the blood [15] (Fig. 1, Table 1). Severe hypertriglyceridemia that appears at a young age is often due to monogenic causes [16•]. However, the vast majority of hypertriglyceridemias in the adult population have a complex polygenic basis where the cumulative effect of multiple independent variants, together with non-genetic factors, elevates triglyceride levels [10] (Fig. 2). During the last decade, genome-wide association studies (GWASs) have uncovered more than 300 independent genetic loci [17–22, 23••, 24–33] associated with plasma triglyceride levels, opening new avenues for early prediction of hypertriglyceridemia and the development of more effective treatments [34].

**Table 1:** Genetic disorders leading to hypertriglyceridemia

Genetic abnormality	Resulting defect	Resulting phenotype	Clinical symptoms	Prevalence
Autosomal recessive mutation in *LPL*, *APOC2*, *APOA5*, *GPIHBP1*, or *LMF1*	Accumulation of chylomicrons in plasma due to reduced LPL-mediated clearance	Familial chylomicronemia syndrome	Severe hypertriglyceridemia; milky appearance of plasma; history of abdominal pain and pancreatitis; lipemia retinalis; eruptive xanthomas	1:100,000–1:1,000,000
Autosomal recessive mutation in *GPD1*	Accumulation of glycerol-3-phosphate in hepatocytes, leading to increased TG synthesis and secretion via VLDL	Transient infantile hypertriglyceridemia	Transient, moderate to severe hypertriglyceridemia in infancy; hepatomegaly; hepatic steatosis	< 1:1,000,000
Autosomal recessive mutation in *AGPAT2*, *BSCL2*, *CAV1*, or *CAVIN1*	Inability to store TG in adipocytes	Congenital generalized lipodistrophy	Generalized fat loss usually at birth or during the first year of life; marked muscularity; moderate to severe hypertriglyceridemia; insulin resistance	1:1,000,000–1:10,000,000
Autosomal dominant mutation in *LMN*, *PPARG*, *PLIN1*, or *AKT2*; autosomal recessive mutation in *CIDEC* or *LIPE*	Selective inability to store TG in subcutaneous adipocytes in various areas of the body	Familial partial lipodystrophy	Fat loss in limbs and gluteal region observed in childhood or puberty; excess fat in the face, neck, and intra-abdominal region; insulin resistance	1:100,000–1:1,000,000
Autosomal recessive E2 allele or dominant E3 or E4 allele in *APOE*, together with secondary factors and likely a polygenic component	Accumulation of RLP in plasma due to reduced APOE-mediated clearance	Familial dysbetalipoproteinemia	Hypertriglyceridemia and hypercholesterolemia; low HDL; VLDL/triglyceride ratio > 0.3	1:1000–2:100
Heterozygous variant in *LPL*, *APOC2*, *APOA5*, *GPIHBP1* or *LMF1*, and/or polygenic predisposition + secondary factors	Increased secretion of VLDL by the liver	Familial hypertriglyceridemia	Moderate hypertriglyceridemia; low HDL; cholesterol/triglyceride ratio < 0.2; obesity and insulin resistance often concomitant	1:100–10:100
Heterozygous variant in *LPL*, *USF1*, *APOA1-C3-A4-A5*, *APOE*, *TNFRSF1B*, or LDLR and/or polygenic predisposition + secondary factors	Decreased clearance of RLP by the liver	Familial combined hyperlipidemia	Hypertriglyceridemia, hypercholesterolemia, or both, which segregate with first-degree relatives; family history or premature cardiovascular disease	5:1000–2:100

*LDL-c*, low-density lipoprotein; *LPL*, lipoprotein lipase; *RLP*, remnant lipoprotein; *TG*, triglyceride; *VLDL*, very low-density lipoprotein

In this review, we will outline recent progress in the understanding of the genetic underpinnings of hypertriglyceridemia.

## Monogenic Hypertriglyceridemia

### Familial Chylomicronemia Syndrome

In familial chylomicronemia syndrome (FCS), an autosomal recessive or compound heterozygous variant in a critical gene regulating chylomicron catabolism is found [1, 8, 16•], leading to high chylomicron levels and low levels of other lipoprotein species in the plasma [1, 16•]. Chylomicrons deliver triglycerides absorbed from the diet to adipose tissue, skeletal muscle, and cardiac muscle where the enzyme lipoprotein lipase (LPL) breaks down the triglycerides (Fig. 1) [15, 35]. In more than 80% of FCS cases, the disease is caused by an autosomal recessive variant in the *LPL* gene [1, 36]. The remaining FCS cases are due to autosomal recessive or compound heterozygous variants in *APOA5*, *APOC2*, *GPIHBP1*, or *LMF1* that all affect LPL function and maturation and thereby interfere with chylomicron levels [8, 15]. More specifically, APOA5 and APOC2 are activators of LPL, LMF1 is needed for the maturation and transport of active LPL, and GPIHBP1 anchors LPL on the surface of capillary endothelial cells.

The prevalence of FCS is in the range of 1:100,000 to 1:1,000,000 [1, 15, 37]. The patients typically have severe hypertriglyceridemia (> 10 mmol/L) and high fasting chylomicron levels from a young age [16•, 36]. As chylomicrons are large molecules, they can block pancreatic capillaries, which together with severe hypertriglyceridemia increase the risk of pancreatitis [1]. Thus, the patients often have a history of acute or recurrent pancreatitis. Other common comorbidities include lipemia retinalis, eruptive tuberous or palmar crease xanthomas, enlarged liver and spleen, and focal neurological symptoms [8, 16•].

### Transient Infantile Hypertriglyceridemia

Transient infantile hypertriglyceridemia is caused by an autosomal recessive variant in the glycerol-3-phosphate dehydrogenase 1 (*GPD1*) gene that causes defective lipid synthesis and a high rate of triglyceride secretion by the liver. Moderate to severe hypertriglyceridemia is present at birth, but triglyceride levels normalize during growth in childhood and adolescence [38], compatible with the fact that the rate of hepatocyte triglyceride secretion is higher during the neonatal period than in adults.

### Congenital Lipodystrophy

Severe hypertriglyceridemia is also commonly observed in patients with monogenic lipodystrophy syndromes, characterized by a complete or partial loss of body fat due to an inability to store fat in the adipose tissue [39, 40]. The most common types are congenital generalized lipodystrophy and familial partial lipodystrophy, caused by autosomal recessive or dominant variants in critical genes involved in lipid droplet and adipocyte function [40–42] (Table 1, Fig. 1). In lipodystrophy, triglycerides are increasingly deposited in non-adipose organs where they induce lipotoxicity. The patients show insulin resistance, diabetes, and fatty liver disease [41, 42]. Monogenic lipodystrophies are rare: the prevalence estimates range from 1:20,000 [43, 44] to less than 1:10,000,000 [40, 41]. Lipodystrophies are not always due to direct genetic reasons — they may be acquired as a result of certain medications and autoimmune reactions [45]. While it is possible that individuals may be genetically predisposed to develop certain types of acquired lipodystrophy, this remains unproven.

## Familial Dysbetalipoproteinemia

Familial dysbetalipoproteinemia (FDBL) is due to autosomal homozygous recessive E2 allele in the apolipoprotein E (*ApoE*) gene, or in ~10% of FDBL cases, a dominant E3 or E4 allele [46–48]. The underlying genetic variants are not deterministic — only 5–10% of individuals homozygous for the *ApoE2* variant develop FDBL [46, 49, 50]. The presence of secondary factors, such as diabetes or high alcohol intake, is generally required for the disease to manifest. Furthermore, there may be a polygenic component to FDBL, consisting of the cumulative effect of multiple genetic risk variants common in the population [51].

APOE functions as a ligand for remnant lipoproteins (RLP), i.e., the remnants of chylomicrons and VLDL, that bind to LDL receptors and related proteins in the liver — a process that removes RLP from the circulation [52, 53] (Fig. 1). Thus, as a result of defective APOE, the patients have reduced clearance of RLP. As high RLP levels are strongly atherogenic, FDBL can lead to premature cardiovascular disease [46, 47, 54]. Patients with FDBL show both elevated triglyceride and total cholesterol levels, generally in the range of 7 to 10 mmol/L, as well as decreased HDL concentration and a VLDL/triglyceride ratio greater than 0.3 [49, 55, 56]. Palmar xanthomas are common [1, 56]. The prevalence of FDBL may be up to 1–2% in the general population, but the condition is often undiagnosed [54, 57].

## Familial Hypertriglyceridemia and Combined Hyperlipidemia

Familial hypertriglyceridemia (FHT) and familial combined hyperlipidemia (FCH) are common conditions that sometimes cluster in families. Early studies of extended families inferred that FHT and FCH may show autosomal dominant inheritance, attributing these conditions to monogenic mutations [58]. However, subsequent investigations revealed that FHT and FCH are of polygenic origin, resulting from the cumulative effect of multiple common variants with small effects on triglyceride levels [34, 51, 59] and/or rare (minor allele frequency < 1%) heterozygous variants with larger effect sizes on triglyceride levels [59, 60] (Fig. 2). The genes for which heterozygous variants have been implicated in FHT include *LPL* [61, 62], *APOA5*, *APOC2*, *GPIHBP1*, and *LMF1* [63] which influence LPL maturation and function. The genes implicated in FCH include *LPL* [64–66], the gene-cluster *APOA1-C3-A4-A5* [67–70], *APOE* [71], *USF1* [72], *TNFRSF1B* [73], and *LDLR* [74] which affect LPL maturation and function, clearance of RLP, or VLDL secretion from the liver. Both FHT and FCH are usually accompanied by secondary factors that exacerbate the elevated triglyceride levels, such as diabetes or high alcohol intake [75]. While we refer to FHT and FCH as “familial” conditions in order to be consistent with previous literature, we note that this term is misleading as it implies a monogenic disorder [76].

In FHT, triglyceride levels are usually moderately elevated and LDL cholesterol and ApoB levels are normal or low, HDL cholesterol levels are decreased, and cholesterol:triglyceride ratio is below 1:5 [12]. It has been estimated that FHT may be present in up to 5–10% of the adult population. FCH is less common, with an estimated prevalence of 0.5–2% of the adult population [77], and more variable in terms of phenotypic expression. It involves hypertriglyceridemia, hypercholesterolemia, or both that segregate with first-degree relatives [11, 78, 79••, 80]. It is important to distinguish between FHT and FCH, because the latter is generally more atherogenic and associated with a severe increase in cardiovascular risk. Thus, the treatment also needs to be more aggressive [81].

FHT and FCH differ in etiology. FHT is primarily caused by increased secretion of VLDL particles by the liver, whereas FCH results primarily from the accumulation of RLP molecules due to decreased RLP clearance (Fig. 1). Increased RLP levels are strongly atherogenic: RLP may enter the arterial wall and become engulfed by lipid-laden macrophages, also called foam cells. If foam cells erupt, the cholesterol embodied by RLP is released and may accumulate in the arteries. Furthermore, high levels of plasma RLP result in the release of low-grade inflammation molecules, accumulation of free radicals, injuries of the endothelium, and increased endothelium permeability, causing edema and necrosis [52, 82].

## Polygenic Hypertriglyceridemia in the GWAS Era

In the general population, genetic predisposition to moderate hypertriglyceridemia usually reflects the cumulative effect of multiple independent genetic variants on triglyceride levels [79, 83–85]. An increase in triglyceride-increasing allele load is associated with higher levels of triglycerides, and this genetic predisposition is exacerbated by secondary factors such as diet, alcohol, certain medications [1, 16•, 86], and concomitant metabolic conditions such as obesity, fatty liver disease, and diabetes [87]. In twin and family studies, the estimated heritability of triglyceride levels — the proportion of variability explained by genetic variation — has ranged between 40 and 60% [88], while the remaining variability is attributed to non-genetic factors.

An approach that has been found very successful for identifying genetic loci associated with elevated triglyceride levels in the general population is to perform a genome-wide association study (GWAS). In GWAS, genetic variants across the genome are screened for their association with triglyceride levels in a standardized manner, and variants reaching genome-wide significant P values (P < 5 × 10^−8^, corresponding to Bonferroni correction for 1,000,000 tests) are claimed as robustly associated with triglyceride levels. Thus far, > 10 GWAS or gene exome-wide screens of triglyceride levels have been performed, which have included up to 600,000 individuals and identified more than 300 genetic loci associated with triglyceride levels with genome-wide significance [17–22, 23••, 24–33, 89]. The majority of these loci have been identified in individuals of European genetic ancestry. A handful of loci have been identified in individuals of African and Asian or Hispanic genetic ancestry [23••, 24–27, 90].

The vast majority (> 90%) of the variants identified thus far are common in the population (minor allele frequency > 1%) and have effect sizes ranging from 0.01 (~2 mg/dL) to 0.26 (~42 mg/dL) standard deviation increase in plasma triglyceride levels per allele. The GWASs have also identified some rare variants with effect sizes up to 1.27 (~205 mg/dL) standard deviation increase in triglyceride levels per allele. Identification of rare genetic variants is expected to be improved through expanded sample sizes and the application of whole-exome and whole-genome sequencing methods that are able to capture rare variants not covered by commercial genotyping arrays [89].

The GWASs performed thus far have implicated several genes already known to be involved in monogenic and familial hypertriglyceridemias, identifying associations of common variants proximal to *LPL*, *APOE*, *APOC2*, and the APOA1-C3-A4-A5 cluster with triglyceride levels [23••]. Pathway enrichment analyses applied on the GWAS summary results have demonstrated that polygenic hypertriglyceridemia is enriched with known mechanisms related to triglyceride metabolism, such as the regulation of LPL, acylglycerol homeostasis, cholesterol transport and storage, and triglyceride-rich lipoprotein metabolism, transportation, and catabolism. However, the GWASs have not only identified already known genes and mechanisms but have also highlighted multiple novel genes that have not been previously known to be involved in the regulation of blood triglyceride levels. This has provided new insights into the biology underlying hypertriglyceridemia and opened avenues for the development of more effective triglyceride-lowering drugs by targeting the implicated genes [91]. The vast majority of the variants identified in GWAS are located in intronic or intergenic regions and the causal genes remain mostly unconfirmed. Pinpointing the causal gene or genes in each associated genetic region will require extensive follow up in experimental studies in vitro and/or in vivo to validate the link between the associated genetic variant or region and modified function of the candidate gene.

To study the combined influence of the triglyceride-increasing genetic loci identified in GWAS on hypertriglyceridemias, genetic variants have been combined into polygenic scores [92] which have been reported to explain up to 19.6% of the variability in triglyceride levels in the general population [93]. Studies applying polygenic scores on FHT and FCH have revealed that the scores are highly enriched in the affected patients, indicating a polygenic basis for these conditions [78, 85]. Polygenic scores have also been applied to try predict hypertriglyceridemia in the general population; however thus far without substantial success. It has become clear that the scores will need to be used in combination with non-genetic factors to increase predictability [94].

In recent years, the genetic loci identified in GWAS have been utilized in Mendelian randomization studies to assess the causal influences of high triglyceride levels on disease traits. Mendelian randomization studies build on the random allocation of genotypes at conception, which makes it possible to assign individuals according to higher or lower genetically determined triglyceride levels in a randomized manner, equivalent to a randomized clinical trial. This reduces the influence of confounding and reverse causality on observational results. Mendelian randomization studies have indicated that higher triglyceride levels are causally associated with higher risk of coronary heart disease and hypertension in the general population [95, 96•].

## Future Perspectives

Hypertriglyceridemia is usually asymptomatic and therefore often undiagnosed and untreated. This elevates the risk for early cardiovascular disease onset and pancreatitis. Being able to early identify the individuals who are at high risk to develop severe hypertriglyceridemia would be valuable, as these individuals could then be screened further and treated to reduce the risk of cardiovascular events and pancreatitis later in life.

Despite recent advances in genetic testing, genetic tests are not yet commonly applied in to identify hypertriglyceridemias [97]. In the coming years, decreasing costs may make these tests more readily available for early genetic screening and counseling. The success of GWAS in identifying genetic loci associated with triglyceride levels have raised interest towards the use of polygenic scores for early prediction and prevention of severe hypertriglyceridemia. However, even though polygenic scores can now explain up to one-fifth of the variability in triglyceride levels in the population [93], the scores have not yet shown value for clinical practice. Furthermore, as GWASs have mostly been performed in adults of European ancestry, the polygenic scores developed thus far are not directly generalizable to individuals of other genetic ancestries. In the coming years, the predictive performance of polygenic scores may be increased through the discovery of additional genetic loci associated with triglyceride levels in diverse genetic ancestries, and by combining polygenic scores with information on non-genetic factors.

Hypertriglyceridemia can be treated pharmacologically with statins, fibrates, niacin and omega-3 acids, and non-pharmacologically with lifestyle changes, such as calorie-restricted diets and exercise [1, 8]. The goal of the treatments is to reduce the risk of cardiovascular disease and pancreatitis. The triglyceride-lowering treatments are currently applied in “a one-size-fits-all” manner to all individuals [98]. However, the pathogenic mechanisms that elevate triglyceride levels are heterogeneous between individuals. The discoveries in GWAS may provide opportunities for tailoring triglyceride-lowering treatments to specific individuals or groups by identifying the specific genetic mechanisms that are affected.

Genetic findings have also opened avenues for the development of more effective triglyceride-lowering drugs, by targeting the identified genes and pathways [91]. For instance, whole-exome sequencing studies solidified the causal role of *APOC3* in the regulation of triglyceride levels and risk of coronary artery disease. Antisense oligonucleotides that target *APOC3* mRNA were recently approved by the European Medicines Agency for the treatment of FCS. As another successful example, GWASs have identified both common and rare variants in the angiopoietin-like 3 (*ANGPTL3*) gene associated with plasma triglyceride levels. The *ANGTPL3* variants inhibit LPL activity, and recent Phase 1 trials for monoclocal antibodies that similarly inhibit *ANGTPL3* demonstrated that the antibodies reduce triglyceride levels in hypertriglyceridemic patients [99]. The antibodies have previously been approved by the U.S. Food and Drug Administration for the treatment of homozygous familial hypercholesterolemia. Finally, GWASs for plasma triglycerides have implicated several genes involved in hepatic lipogenesis, a previously underappreciated pathway in the regulation of plasma triglyceride levels [91]. However, most of the identified genes are not associated with risk of cardiovascular disease, suggesting that hepatic lipogenesis may not be a sufficient target to prevent cardiovascular events [91].

At present, the causal genes and their functions remain unknown for the vast majority of the loci identified in GWAS, which hinders translational efforts. The increased use of whole-exome and whole-genome sequencing may lead to enhanced identification of rare coding variants that lead to loss or gain of gene function and show large effect sizes on triglyceride levels. Such variants have particularly high potential for drug targeting [89].

## Conclusions

Hypertriglyceridemia can have a monogenic or polygenic basis. In monogenic hypertriglyceridemias, which are extremely rare, homozygous genetic variants leading to dysfunctional proteins involved in triglyceride metabolism lead to severe hypertriglyceridemia. In the vast majority of cases, however, hypertriglyceridemia is of complex polygenic origin, whereby the cumulative effects of multiple genetic variants, together with non-genetic factors, contribute to elevated levels of triglycerides. Over recent years, familial hypertriglyceridemia and combined hyperlipidemia, previously considered monogenic, have been found to be mainly polygenic conditions.

In GWAS performed to date, > 300 independent genetic loci have been found associated with plasma triglyceride levels, revealing new biology and opening avenues for the development of more effective treatments against hypertriglyceridemia. Successful examples of GWAS-identified targets for triglyceride-lowering drugs include *APOC3* and *ANGPTL3*. However, for the vast majority of identified loci, the causal genes remain unknown, warranting extensive efforts to experimentally validate the causal genes and their functions in vitro and in vivo.

The GWAS findings may allow the development of early screening tools for severe hypertriglyceridemia. At present, nearly all loci currently pertain to European ancestry, and more efforts are required to increase knowledge on the genetic basis of hypertriglyceridemia in non-European ancestries. Another emerging possibility is to use genetic information to better tailor triglyceride-lowering treatments for individual patients, based on the specific genetic mechanisms that are affected.
